# Measurement of Cortical Atrophy and Its Correlation to Memory Impairment in Patients With Asymptomatic Carotid Artery Stenosis Based on VBM-DARTEL

**DOI:** 10.3389/fnagi.2021.620763

**Published:** 2021-07-05

**Authors:** Peijiong Wang, Husule Cai, Rutao Luo, Zihao Zhang, Dong Zhang, Yan Zhang

**Affiliations:** ^1^Department of Neurosurgery, Beijing Tiantan Hospital, Capital Medical University, Beijing, China; ^2^China National Clinical Research Center for Neurological Diseases (NCRC-ND), Beijing, China; ^3^Center of Stroke, Beijing Institute for Brain Disorders, Beijing, China; ^4^Beijing Key Laboratory of Translational Medicine for Cerebrovascular Disease, Beijing, China; ^5^Department of Neurosurgery, Beijing Children’s Hospital, Capital Medical University, National Center for Children’s Health, Beijing, China; ^6^State Key Laboratory of Brain and Cognitive Science, Beijing MRI Center for Brain Research, Institute of Biophysics, Chinese Academy of Sciences, Beijing, China

**Keywords:** asymptomatic carotid stenosis, memory deficiency, VBM analysis, cerebral gray matter atrophy, 7T-MRI

## Abstract

**Objective:**

Severe carotid artery stenosis (CAS) can lead to atrophy of gray matter (GM) and memory impairment; however, the underlying mechanism is unknown. Thus, we aimed to identify memory impairment and GM atrophy and explore the possible correlation between them in patients with asymptomatic severe CAS.

**Methods:**

Twenty-four patients with asymptomatic severe CAS and 10 healthy controls completed the mini-mental state examination (MMSE) and clinical memory scale (CMS) and underwent 7T magnetic resonance imaging (MRI) scan. Field intensity inhomogeneities were corrected. Images were processed using VBM8, and GM images were flipped. First, 11 flipped and 10 non-flipped images of patients with unilateral CAS and 5 flipped and 5 non-flipped images of controls were pre-processed using DARTEL algorithm and analyzed using an analysis of variance (ANOVA). Second, flipped and non-flipped images of unilateral patients were similarly pre-processed and analyzed using the paired *t*-test. Third, pre-processed non-flipped GM images and CMS scores of 24 patients were analyzed by multiple regression analysis. Nuisance variables were corrected accordingly.

**Results:**

Basic information was well matched between patients and controls. MMSE scores of patients were in the normal range; however, memory function was significantly reduced (all *P* < 0.05). GM volumes of patients were significantly reduced in the anterior circulation regions. The stenosis-side hemispheres showed greater atrophy. GM volumes of the left pars opercularis, pars triangularis, and middle frontal gyrus were strongly positively correlated with the total scores of CMS (all *r* > 0.7, *P* = 0.001). Additionally, the left middle frontal gyrus was strongly positively correlated with associative memory (*r* = 0.853, *P* = 0.001). The left pars opercularis was moderately positively correlated with semantic memory (*r* = 0.695, *P* = 0.001).

**Conclusion:**

Patients with asymptomatic CAS suffer from memory impairment. Bilateral anterior circulation regions showed extensive atrophy. The hemisphere with stenosis showed severer atrophy. Memory impairment in patients may be related to atrophy of the left frontal gyrus and atrophy of different regions may result in different memory impairments.

## Introduction

Carotid artery stenosis (CAS), a type of vascular degenerative disease, can cause cerebral ischemic events, such as amaurosis, transient ischemic attack (TIA), and stroke, which threaten life and health. Several large-sample studies, such as the Cardiovascular Health Study and the Framingham Study, have revealed that CAS can lead to cortical atrophy (Newman et al., 2005; Romero et al., 2009) and the MART-MR study confirmed progressive loss of gray matter (GM) in patients with severe CAS during long-term follow-up (Muller et al., 2011). However, these studies focused primarily on whole brain GM volumes and left specific areas of atrophy unexplored. Despite some recent researches reporting that cortical atrophy mainly occurs in cerebral areas supplied by the anterior circulation (Asllani et al., 2016; Avelar et al., 2016; Marshall et al., 2017), few studies have investigated the location and characteristics of atrophy, especially difference in cerebral atrophy between the hemisphere affected by severe stenosis and the contralateral hemisphere.

Moreover, cognitive impairment was detected in around 50% of patients with severe CAS (Pettigrew et al., 2000; Smith, 2017), and memory decline, one of the most common complaints, was reported in 25.5% patients approximately (Luo et al., 2018). Furthermore, thinning of the cortex is considered a potential biomarker, which has shown to be associated with cognitive impairment in aging, neurodegenerative disease, and small vascular disease (Seo et al., 2010; Kim et al., 2014; Pettigrew et al., 2016; Weston et al., 2016). All these studies raise the possibility that cortical thinning in CAS patients may contribute to memory decline; however, this has not been clarified. Furthermore, locating the specific correlating cortex may allow a better understanding of the mechanism of memory impairment in CAS patients.

Thus, we acquired 7T-MRI T1 images and cognitive scores from patients with severe asymmetric CAS and healthy controls. This study aimed to show the changes in memory function and gray matter volumes of patients to delineate whether cerebral atrophy in patients was asymmetric between the stenosis-side hemisphere and the contralateral hemisphere and to better understand the correlation between memory impairment and GM atrophy.

## Materials and Methods

This study was approved by the ethics committee of Beijing Tiantan Hospital, Capital Medical University. All patients and healthy volunteers were voluntarily involved in this study and provided consent. The inclusion criteria of CAS patients were as follows: (1) severe CAS (grade of stenosis ≥70%), based on either or both computed tomography angiography (CTA) and digital subtraction angiography (DSA), following the guidelines for CAS (criteria of the North American Symptomatic Carotid Endarterectomy Trial) (Barnett et al., 1998); and (2) asymptomatic CAS, where no cerebral ischemic events (amaurosis, TIA, and stroke) had occurred during the last 6 months. The exclusion criteria of CAS patients were as follows: (1) contraindications to magnetic resonance imaging (MRI), such as claustrophobia or metal implants in the body; (2) history of any other cognitive impairment disease, such as Alzheimer’s disease; and (3) history of surgery of the carotid artery. The inclusion criteria of healthy volunteers were as follows: (1) no carotid stenosis; (2) no history of cerebral disease; and (3) no history of cognitive impairment. To match healthy volunteers with patients, volunteers were recruited online using random stratified sampling according to age, sex, and educational background.

A total of 24 patients with severe asymptomatic CAS and 10 healthy volunteers were recruited from April 2016 to August 2018. All patients and healthy volunteers completed cognitive scales and underwent 7T-MRI scan. Baseline information obtained for the study included age, sex, and educational background.

### Cognitive Examination

The cognitive function of patients and volunteers were assessed using the mini-mental state examination (MMSE) and clinical memory scale (CMS), which was completed several days before the MRI scan. All tests and reports were conducted by doctors.

The MMSE was used to estimate general cognitive function and detect moderate and severe cognitive impairment. MMSE consists of 30 items, which include evaluation of temporal and spatial orientation, verbal memory, attention and calculation, short-term memory, object naming, retelling, reading comprehension, language comprehension, speech articulation, and graphic drawing, among others. Reference scores for cognitive impairment are defined as ≤18 for illiteracy, ≤21 for primary school education, and ≤25 for middle school education and above.

Clinical memory scale was employed to assess memory function. CMS was adapted by the Institute of Psychology of Chinese Academy of Sciences and is composed of five tests, which include directed memory, associative memory, meaningless graphic recognition, free image recall, and portrait characteristic recall. After the test, original scale scores were converted into scores according to age and educational background, according to user guide. Then, the five scores were summed up to obtain the total scale score. The total scale score was transformed into a memory quotient (MQ) according to the user guide. The grades of memory function were defined as follows: MQ ≥ 130 as super-excellence, 120–129 as excellence, 110–119 as upper level, 90–109 as moderate level, 80–89 as lower level, 70–79 as poor, and MQ < 70 as very poor. Directed memory test and associative learning tests were used to assess the semantic memory function, and these two scores were summed up to obtain a semantic memory score.

### 7T-MRI Acquisition

MRI data were acquired at the Beijing Brain MRI Center using the 7T-MRI investigational system (Magnetom 7T, Siemens Healthineers, Erlangen, Germany), which was equipped with a Nova 32 channel phased-array head coil. We chose T1-MPRAGE as researching MRI sequence with the following parameters: TR 2200 ms, TE 3.01 ms, flip angle 7°, FOV 224 × 224 mm^2^, matrix 320 × 320, slice thickness 0.70 mm, and voxel size 0.7 × 0.7 × 0.7 mm^3^.

### Processing and Analysis of MRI Images

Because 7T T1-MPRAGE images are not acquired with additional gradient echo (GE) images to correct intensity inhomogeneities of the bias field, we followed a two-step pre-processing pipeline. First, the non-parametric non-uniform intensity normalization (N3) algorithm was used before VBM processing. This step was implemented in the FreeSurfer software and followed the optimal parameters (proto-iters = 1000, distance = 15, *n* = 1), proposed by Lüsebrink et al. (2013) Second, two parameters were adapted for VBM processing to correct any remaining intensity inhomogeneities using the following parameters: bias regularization changed from the default of “very light” to “extremely light, bias FWHM cutoff changed from the default of 60 to 30 mm (Lüsebrink et al., 2013).

After processing using the N3 algorithm, three-dimensional (3D) T1 images were processed automatically using voxel-based morphometry (VBM) analysis in VBM8 software running in SPM8 under MATLAB 2012a. During this procedure, two parameters were adapted as above, and a symmetric tissue probability map (TPM) provided by Kurth et al. (2015) was used instead of the default unsymmetrical TPM. The T1 images were spatially normalized and then segmented into gray matter (GM), white matter (WM) and cerebrospinal fluid (CSF). Meanwhile, the absolute volumes (ml) of GM, WM, and CSF were calculated. Total intracranial volume (TIV) was calculated as the sum of the absolute volumes of GM, WM, and CSF. GM images of all subjects were then flipped in the right–left orientation to obtain corresponding mirror GM images in SPM8 using image calculation.

#### Patients vs. Controls

We defined the left cerebral hemisphere as the stenosis-side hemisphere and the right hemisphere as the contralateral hemisphere. Thus, flipped GM images of patients with right CAS should be involved, and an equal proportion of flipped images of controls should be involved for comparison. The flipped images of controls were selected at random. In total, there were 11 flipped GM images of patients with right CAS, 10 non-flipped GM images of patients with left CAS, and 5 flipped and 5 non-flipped GM images of healthy volunteers. All these images were processed using the DARTEL algorithm, modulated by flow field files and smoothed at an 8-mm full width at half maximum. The GM template was used to make a mask to limit analysis to the cortex. The grayscale threshold was a minimum of 0.3. Finally, the smoothed GM images were analyzed using an analysis of variance (ANOVA) of all factors (a 2 × 2 ANOVA) in SPM8 software to verify the impact of CAS on cerebral atrophy and eliminate the effect of sides. The nuisance variables TIV, sex, age, and educational background were used for correction. Statistical significance was defined as *P* < 0.05 using false discovery rate (FDR) correction and a minimum of 200 contiguous voxels. After comparison, atrophic cerebral areas on the left hemisphere demonstrated the effect of CAS on the stenosis-side hemisphere, and areas on the right hemisphere demonstrated the effect of CAS on the contralateral hemisphere. This enabled us to determine whether atrophy caused by unilateral CAS was unilateral or bilateral and where it was located, while eliminating the impact of laterality.

#### Stenosis-Side Hemisphere vs. Contralateral Hemisphere

**FIGURE 1:**
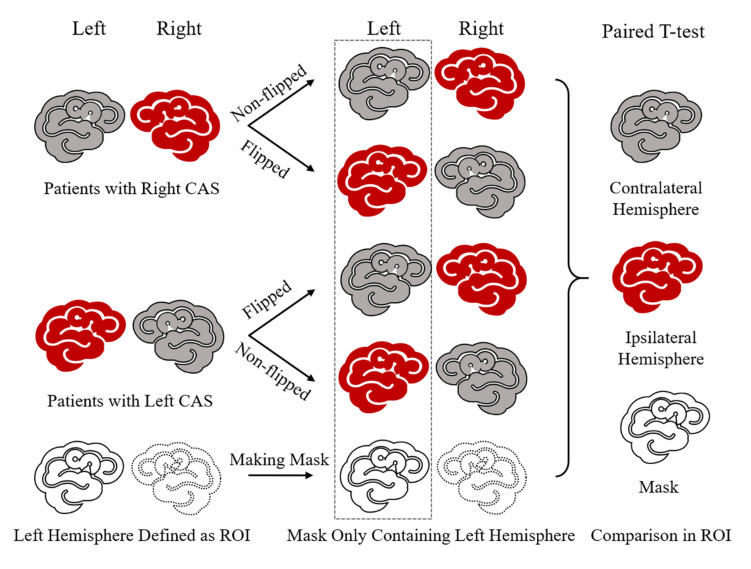
Diagram of stenosis-side hemisphere vs. contralateral hemisphere. First, in each unilateral CAS patient, ipsilateral hemisphere and contralateral hemisphere cannot be paired and compared directly. So, the GM image of each patient needs to be flipped in the right–left orientation and then each patient had one non-flipped image and one paired flipped image. In this way, each patient had a stenosis hemisphere and a contralateral image on the left side when his/her non-flipped image and flipped image were paired together. Thus, patients’ non-flipped images and flipped GM images were analyzed by paired *t*-test following mode of contralateral hemisphere vs. stenosis-side hemisphere on the left side defined by left-hemisphere mask.

We defined the left cerebral hemisphere as the region of interest (ROI). The non-flipped and flipped GM images of 21 patients were processed using the DARTEL algorithm, and then images were modulated by flow field files and smoothed at an 8-mm full width at half maximum. The left-hemisphere mask was made using the GM template with grayscale threshold >0.3, which limited the analysis to the left hemisphere. Finally, smoothed non-flipped and flipped GM images were analyzed using the paired *t*-test to compare the contralateral hemisphere with the stenosis-side hemisphere on the left side defined by the left-hemisphere mask. The statistical significance was defined as *P*_FDR_ < 0.05 and a minimum of 100 contiguous voxels. The diagram is shown in Figure 1.

#### Memory-Related Cortex

The non-flipped GM images of 24 patients were processed using the DARTEL algorithm to create templates and then these images were modulated and smoothed at an 8-mm full width at half maximum. The mask was made using the GM template with a grayscale threshold of 0.3. The smoothed GM images and CMS scores, including the total scores, the five subtest scores, and semantic memory scores, were analyzed successively using multiple regression analysis in SPM8 software. The nuisance variables TIV, sex, and degrees of carotid stenosis on each side were used for correction. Age and educational background were not used for correction because they had already been adjusted for during scoring. Statistical significance was defined as *P*_FDR_ < 0.05 and a minimum of 100 contiguous voxels.

If correlated regions were detected, these were each defined as an ROI and a corresponding mask was created. A customized script referring to Kurth’s “extract” script was used to extract the volume (mm^3^) from each GM image in MATLAB R2012a, and each volume was divided by corresponding TIV to obtain the relative volume (Kurth et al., 2015). Relative volumes and CMS scores were analyzed by Pearson correlation analysis in SPSS (Windows version 23.0, IBM).

### Statistical Analysis

Statistical analyses were conducted in SPSS (Windows version 23.0, IBM). Continuous variables are shown as means ± standard deviations, and categorical values are described as percentages (numbers). Absolute volumes of GM, WM, CSF, and TIV were used as references to correct results during analysis but were not used as explicit values for data comparison. We focused mainly on the relative volumes, which were obtained by dividing the absolute volumes by the corresponding TIV. A chi-square test was used to compare categorical data. Student’s *t*-test and rank sum test were used to compare continuous data. Pearson correlation analysis was used to correlate relative volumes of correlated cerebral areas with CMS scores, and the Pearson correlation coefficient (*r*) indicated the strength of correlation. Correlation was considered strong when 1 > *r* ≥ 0.7, moderate when 0.7 > *r* ≥ 0.4, and weak when 0.4 > *r* > 0 (Dancey and Reidy, 2007). Statistical significance was defined as *P* < 0.05. VBM results were shown as 3D reconstructed images and slice views using xjView software.

## Results

Baseline information, including age, gender, details of stenosis, and education background, is listed in Table 1, and there were no statistical differences between patients with asymptomatic severe CAS and healthy controls. For cognitive function, MMSE scores of patients with CAS were in normal range and were similar to those of healthy controls (all *P* ≥ 0.05). For CMS, the total scores, MQ, and memory function grade of the patient group were much lower than those of the control group (all *P* < 0.05). Moreover, the scores of the four subtests (i.e., directed memory, associative learning, free image recall, and portrait characteristic recall) were lower in patients with CAS than in healthy controls (all *P* < 0.05). Meaningless graphic recognition test scores were lower in patients than in healthy controls; however, this did not reach statistical significance (*P* = 0.080; Table 2). Compared with healthy controls, the relative GM volumes of patients were significantly lower (*P* = 0.027), and the relative CSF volumes were significantly higher (*P* = 0.001), whereas relative WM volumes were similar between the two groups (*P* = 0.082). The reduction in relative GM volume was approximately 3.1% (Table 3).

**TABLE 1:** Basic information of CAS patients and healthy controls.

	CAS patients (*n* = 24)	Healthy controls (*n* = 10)	*P*-value
Age (years)	63.8 ± 5.6	65.0 ± 5.1	0.585
Gender (male)	15 (62.5%)	6 (60.0%)	1.000
**Carotid stenosis**			
Bilateral	3 (12.5%)	–	–
Unilateral	21 (0.875)	–	–
Left	10 (47.6%)	–	–
Right	11 (52.4%)	–	–
**Contralateral**			
Moderate	5 (23.8%)	–	–
Mild	5 (23.8%)	–	–
None	11 (52.3%)	–	–
**Education**			0.493
None	2 (8.3%)	0 (0.0%)	
Primary school	5 (20.8%)	1 (10.0%)	
High school	14 (58.4%)	7 (70.0%)	
University/above	3 (12.5%)	2 (20.0%)	
			

**TABLE 2:** Cognitive function assessment of CAS patients and healthy controls.

	CAS patients (*n* = 24)	Healthy controls (*n* = 10)	*P*-value
**MMSE**			
Scores	26.2 ± 2.1	27.3 ± 0.9	0.050
Grade of MMSE			1.000
Normal	24 (100%)	10 (100%)	
Impairment	0 (0%)	0 (0%)	
**CMS**			
Directed memory	10.6 ± 5.8	19.6 ± 2.0	0.001
Associative memory	11.1 ± 4.5	22.1 ± 3.0	0.001
Meaningless graphic recognition	11.6 ± 5.1	21.0 ± 2.7	0.001
Free image recall	15.4 ± 8.1	18.8 ± 1.9	0.080
Portrait characteristic recall	15.0 ± 5.5	19.2 ± 3.1	0.037
Semantic memory	41.7 ± 4.7	22.1 ± 9.3	0.001
Total scores	64.3 ± 19.5	99.4 ± 11.2	0.001
MQ	80.4 ± 14.9	108.4 ± 9.3	0.001
**Grade of memory**			0.001
Super-excellence (≥130)	0 (0%)	0 (0%)	
Excellence (120–129)	0 (0%)	1 (10%)	
Upper level (110–119)	1 (4.8%)	3 (30%)	
Moderate level (90–109)	6 (28.6%)	5 (50%)	
Lower level (80–89)	2 (9.5%)	1 (10%)	
Poor (70–79)	6 (28.6%)	0 (0%)	
Very poor (≤69)	6 (28.6%)	0 (0%)	
			

**TABLE 3:** Brain volumes of different parts in CAS patients and healthy controls.

	Healthy controls (*n* = 10)	Total CAS patients (*n* = 24)		Unilateral CAS patients (*n* = 21)		Bilateral CAS patients (*n* = 3)	
		Data	*P*	Data	*P*	Data	*P*
**Absolute volume**							
GM volume/ml	596.0 ± 50.2	527.7 ± 50.14	0.001	534.00 ± 50.06	0.003	483.87 ± 23.68	0.004
WM volume/ml	539.5 ± 71.2	475.77 ± 51.29	0.006	484.60 ± 47.97	0.017	413.99 ± 26.46	0.014
CSF volume/ml	231.1 ± 26.4	244.2 ± 37.9	0.327	245.90 ± 37.07	0.267	232.35 ± 50.44	0.953
TIV volume/ml	1366.1 ± 139.4	1247.7 ± 113.5	0.014	1264.50 ± 110.87	0.014	1130.22 ± 40.98	0.017
**Relative volume**							
GM volume/%	43.69 ± 1.24	42.34 ± 2.17	0.029	42.26 ± 2.16	0.027	42.85 ± 2.63	0.640
WM volume/%	39.39 ± 1.59	38.11 ± 1.80	0.058	38.31 ± 1.56	0.082	36.68 ± 3.06	0.057
CSF volume/%	17.03 ± 0.90	19.28 ± 2.43	0.001	19.28 ± 2.43	0.001	20.47 ± 3.65	0.012
Reduced GM volume/%	–	3.1	–	3.3	–	1.9	–

**FIGURE 2:**
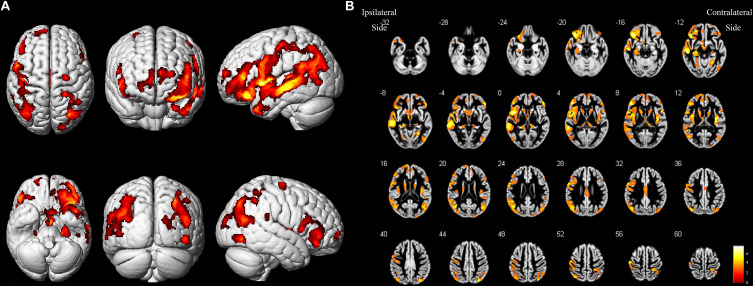
Asymptomatic CAS patients vs. healthy controls. **(A)** Three-dimensional image. **(B)** Slice view. Cortical atrophy was observed in bilateral frontal, temporal, and parietal lobes, but occipital lobe and cerebellum were barely affected. L: left, R: right.

**FIGURE 3:**
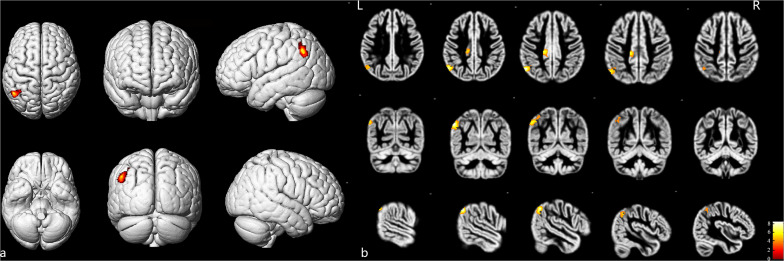
Stenosis-side hemisphere vs. contralateral hemisphere. **(a)** Three-dimensional image. **(b)** Slice view. The stenosis-side hemisphere suffered more severe atrophy on inferior parietal lobule and cingulate gyrus, compared with contralateral hemisphere. L: left, R: right.

As shown in Figure 2 and Table 4, loss of GM volume in patients was not only limited to the stenosis-side hemisphere, but also observed in the contralateral hemisphere. Atrophy was widely distributed in the frontal, temporal, and parietal lobes as well as in smaller regions of the occipital lobes on both the ipsilateral and contralateral hemispheres. Meanwhile, large parts of the occipital lobes and cerebellar lobes were unaffected bilaterally. Furthermore, the stenosis-side hemisphere showed more severe atrophy of the inferior parietal lobule and cingulate gyrus compared with the contralateral hemisphere (Figure 3 and Table 5).

**TABLE 4:** Details of gray matter regions reaching statistical significance in comparison between CAS patients and healthy controls.

Anatomical structure	Brodmann area	MNI coordinates			Voxel size	*P*_FDR_	*t*	*z*
		*x*	*y*	*z*				
***On Stenosis Side***								
Frontal and temporal lobe	21, 22, 13, 40, 11	-42	34.5	6	25300	0.001	7.47	5.27
Limbic lobe	36, 25	−22.5	−24	−7.5	4029	0.001	6.03	4.62
Basal ganglia		−24	−43.5	−10.5	378	0.013	3.72	3.26
Superior frontal gyrus	10, 32	−12	66	15	1262	0.006	4.39	3.70
Cingulate gyrus	23, 24	0	−12	31.5	787	0.010	3.94	3.41
***On contralateral side***								
Inferior frontal gyrus	47, 44, 45	51	36	−3	1766	0.002	5.48	4.34
Limbic lobe	19, 36	22.5	−55.5	−7.5	531	0.002	5.63	4.42
Middle occipital gyrus	19, 37	45	−73.5	−7.5	593	0.004	4.70	3.90
Parahippocampal gyrus	27, 28	22.5	−24	−9	569	0.003	5.10	4.13
Medial frontal gyrus	10, 32	7.5	52.5	13.5	1017	0.005	4.43	3.73
Insular lobe	13	39	−9	10.5	1259	0.001	6.29	4.75
Occipital cuneus	18, 19	18	−94.5	18	369	0.012	3.79	3.30
Superior parietal lobule	39, 40	30	−73.5	45	3406	0.001	5.84	4.53
Superior temporal gyrus	13, 40	45	−37.5	16.5	219	0.004	4.70	3.90
Superior frontal gyrus	6	25.5	−12	67.5	279	0.007	4.22	3.59

*MNI, Montreal Neurological Institute.*

**TABLE 5:** Details of gray matter regions reaching statistical significance in the ipsilateral and the contralateral comparison and memory function-related cortex.

Anatomical structure	Brodmann area	MNI coordinates			Voxel size	*P*_FDR_	*t*	*z*	r*	P*
		*x*	*y*	*z*						
Ipsilateral vs. Contralateral ^‡^										
Left inferior parietal lobule	40	−51	−58.5	39	236	0.001	8.50	5.47	–	–
Left cingulate gyrus	31	−15	−27	42	124	0.004	6.64	4.77	–	–
Memory function related cortex^§^										
General memory										
Left middle frontal gyrus	10	−22.5	46.5	22.5	312	0.022	5.82	4.31	0.841	0.001
Left frontal pars triangularis	46	−42	27	19.5	252	0.022	5.78	4.29	0.724	0.001
Left frontal pars opercularis	44	−60	6	12	473	0.022	6.16	4.46	0.798	0.001
Associative memory										
Left middle frontal gyrus	10	−30.5	51	13.5	137	0.048	6.86	4.75	0.853	0.001
Semantic memory										
Left frontal pars opercularis	44	−58.5	3	15	157	0.015	6.47	4.59	0.695	0.001

*^‡^Paired *t*-test. ^§^Multiple regression analysis.*

*MNI, Montreal Neurological Institute.*

**FIGURE 4:**
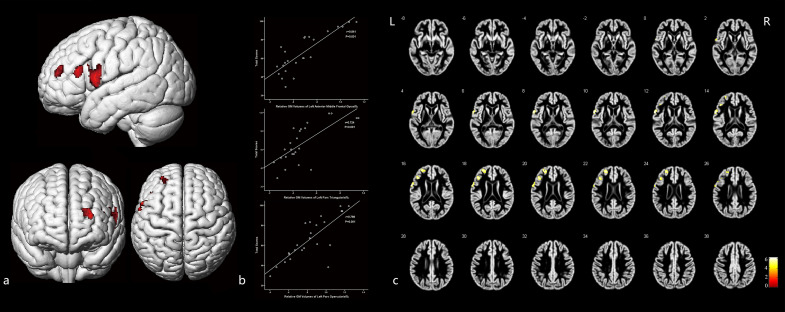
The cortex related with general memory function and their Pearson correlation coefficients. **(a)** Three-dimensional image. **(b)** Correlation between the total scores of CMS and regional relative GM volumes. **(c)** Slice view. GM volumes of the left pars opercularis gyri frontalis inferioris, left pars triangularis gyri frontalis inferioris, and the frontal part of left middle frontal gyrus were positively related with the total clinical memory scores closely. L: left, R: right.

Figure 4 and Table 5 illustrate the positive correlation of GM volumes of the left middle frontal gyrus, left frontal pars triangularis, and left frontal pars opercularis with the total scores of CMS and their Pearson correlations being strong (left middle frontal gyrus: *r* = 0.841, *P* = 0.001; left frontal pars triangularis: *r* = 0.724, *P* = 0.001; left frontal pars opercularis: *r* = 0.798, *P* = 0.001).

**FIGURE 5:**
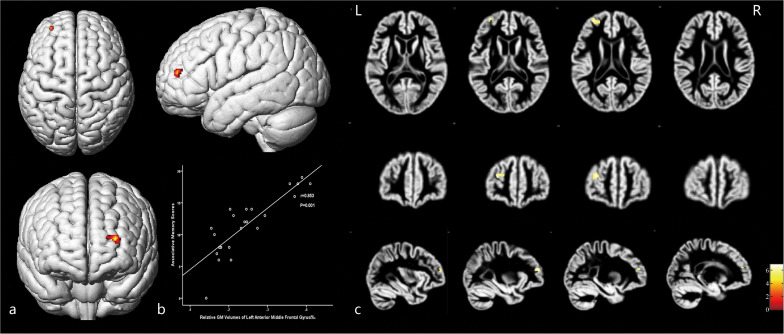
The cortex related to associative memory and their Pearson correlation coefficients. **(a)** Three-dimensional image. **(b)** Correlation between associative scores and regional relative GM volumes. **(c)** Slice view. GM volumes of the anterior medial frontal gyrus cortex were positively related with the associative memory scores. L: left, R: right.

**FIGURE 6:**
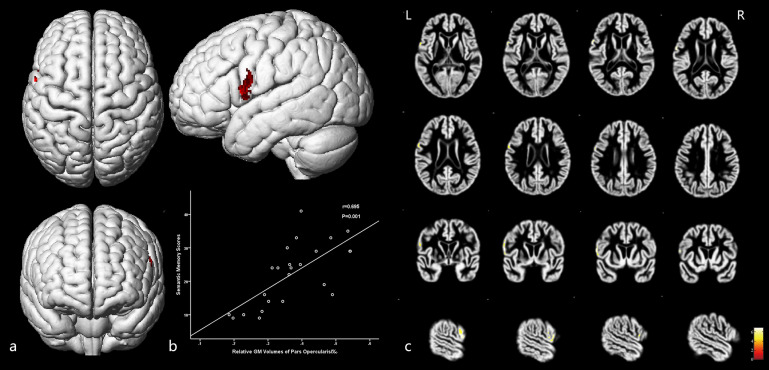
The cortex related to semantic memory and their Pearson correlation coefficients. **(a)** Three-dimensional image. **(b)** Correlation between semantic memory scores and regional relative GM volumes. **(c)** Slice view. GM volumes of the posterior inferior frontal gyrus were positively related with the semantic memory scores. L: left, R: right.

Moreover, GM volumes of the left medial frontal gyrus were strongly positively correlated with the associative memory scores (*r* = 0.853, *P* = 0.001; Figure 5 and Table 5). GM volumes of the left posterior inferior frontal gyrus were moderately positively correlated with the semantic memory scores (*r* = 0.695, *P* = 0.001; Figure 6 and Table 5).

## Discussion

### Cortical Atrophy

This study confirmed cortical atrophy and increased CSF volumes in patients with CAS by analyzing relative GM and CSF volumes and VBM, which were consistent with several previous studies (Newman et al., 2005; Romero et al., 2009; Muller et al., 2011). Large-sample cross-sectional studies, such as the Cardiovascular Health Study and the Framingham Study, reported that moderate and severe CAS leads to brain atrophy, which mainly manifest as enlarged ventricles and sulci and reduced GM volumes (Newman et al., 2005; Romero et al., 2009). Furthermore, a SMART-MR study demonstrated that during a long-term follow-up of 3–9 years, unilateral severe CAS and bilateral CAS caused severe progressive atrophy of the cortex (Muller et al., 2011).

We found that the average reduction in cerebral GM in patients with asymptomatic severe CAS was 3.1%, which is close to 3.7% published by Iris et al. previously (Asllani et al., 2016). Although 3.1% may not seem substantial, the proportion is clinically significant when compared with those of other cognitive diseases. As reported in previous studies, cortical atrophy due to aging is roughly 0.2% every year in middle-aged healthy people (Shaw et al., 2016). The hippocampal cortices has been reported to be 1.2–1.5% thinner in patients with mild cognitive impairment (Das et al., 2012) and 4.7% thinner in patients with Alzheimer’s disease who carry the APOEε4 allele (Harrison et al., 2016). Thus, cortical atrophy in patients with asymptomatic severe CAS deserve clinical attention.

Our VBM analysis revealed that cortical atrophy was widely distributed bilaterally in the frontal, temporal, and parietal lobes; however, the occipital lobes and cerebellum were largely unaffected, which is consistent with the areas of the anterior circulation. This finding is in line with those of several previous studies (Asllani et al., 2016; Avelar et al., 2016; Marshall et al., 2017). This phenomenon may be caused by hypoperfusion, a patent circle of Willis, and susceptibility to hypoperfusion. First, in regard to hypoperfusion, Gabriella et al. (2018) confirmed that carotid artery occlusion can lead to cortical atrophy in a mouse model and found a correlation between cortical atrophy and cerebral hypoperfusion. Marshall et al. (2017) further reported in a human MRI study that anterior circulation hypoperfusion caused by CAS leads to atrophy of the primary motor cortex, and these were positively correlated. Second, a patent circle of Willis may compensate for hypoperfusion by directing blood from the contralateral to the stenosis-side hemisphere, which may result in general hypoperfusion across the whole brain. Previous studies have found that the opening rate of the anterior communicating artery in patients with CAS was significantly higher than that of healthy controls (Lee et al., 2004). Marshall et al. (2017) showed that cerebral blood flow (CBF) of both the anterior and posterior circulation regions of both hemispheres in patients with CAS was lower than that of healthy controls. Third, the same study found that the posterior circulation regions also showed hypoperfusion, whereas the occipital and cerebellar cortices were barely affected, which led them to speculate that the anterior circulation region, but not the but posterior circulation, is susceptible to hypoperfusion (Marshall et al., 2017).

We also found that atrophy of the stenosis-side hemisphere was more severe than that of the contralateral hemisphere. Previous study findings on asymmetrical atrophy are inconsistent. Nickel et al. (2019) did not find significant reductions in cortical thickness in brain regions ipsilateral to the stenosis. Asllani et al. (2016) reported significant reductions in cortical thickness on the stenosis-side hemisphere, but no difference was observed in cortical volumes between the stenosis and the contralateral hemispheres. Marshall and Avelar drew similar conclusions by confirming a greater reduction of cortical volumes of the stenosis-side hemisphere (Avelar et al., 2016; Marshall et al., 2017). Differences in the inclusion and exclusion criteria and algorithms may contribute to the inconsistent findings.

Comparisons between the two hemispheres carry the issue of structural asymmetries of the brain, and detection of brain asymmetries requires methods that can establish accurate spatial correspondence not only across subjects, but also across an individual’s hemispheres. VBM was proven to be capable of capturing gray matter asymmetries with an extremely high voxel-based regional accuracy. Thus, we followed the VBM guideline published by Kurth et al. to analyze the structural asymmetries in cortical atrophy in CAS patients. According to this guideline, regional specificity and accurate spatial correspondence is ensured by the spatial normalization of images into the symmetrical TPM, using the DARTEL algorithm (Kurth et al., 2015). Moreover, we used an explicit brain mask to avoid the blurring of information across hemispheres and to control the possible impact of noise in the data. These modifications were included in our study to maximize the accuracy of our study.

### Cognitive Impairment

In this study, we found that patients with asymptomatic unilateral severe CAS performed poorly in the CMS examination, which indicated memory impairment in patients with CAS. Numerous studies have reported similar levels of memory decline in patients with CAS (Hayashi et al., 2004; Lee et al., 2004; Laura et al., 2012). Scores of directed memory, associative learning, free image recall, and portrait characteristic recall were significantly lower in patients than in healthy controls, which indicated that the corresponding memory functions were impaired. Furthermore, lower scores in the direct memory and associative learning test indicated impaired semantic memory function. Lower scores in the free image recall and portrait characteristic recall test proved that non-verbal memory was also impaired in patients with CAS. Previous studies have also reported similar results (Newman et al., 2005; Laura et al., 2012). However, scores in the meaningless graphic recognition test were lower than those of healthy controls, but not significantly so. Our relatively small sample size may account for this result. Furthermore, this may also indicate that non-verbal memory decline may not have been as prominent as semantic memory impairment.

Mini-mental state examination was used in our research to detect moderate and severe cognitive impairment (Folstein et al., 1978). The MMSE scores of patients with CAS were slightly lower than those of healthy controls; however, the scores were defined as normal, which is consistent with previous studies (Martinić-Popović et al., 2009; Luo et al., 2018). This indicated that cognitive impairment in asymptomatic patients is relatively mild.

### The Relationship Between Memory Impairment and Regional GM Volumes

#### General Memory Function

We found that the GM volumes of the left frontal pars triangularis, left frontal pars opercularis, and the frontal part of the left middle frontal gyrus were strongly positively correlated with the total score of CMS. This indicated that GM atrophy in these areas caused by CAS may be an important factor underlying the memory impairment in patients. Several studies have also found that cerebral atrophy may account for the decline in memory, execution, attention, and other functions, although focus was primarily on whole brain volume rather than specific regional GM volume (Hayashi et al., 2004; Newman et al., 2005; Romero et al., 2009). In this study, we not only identified the specific correlated cortical areas form extensive atrophied areas but also demonstrated how they correlated with memory function. These findings may provide theoretical support and inspiration for clinical practice and further studies.

In cognitive neuroscience, the prefrontal cortex (PFC) has been shown to play an important role in memory functions. D’Esposito and Postle (1999) reported that damage to the PFC may not affect short-term memory but may impair the maintenance and processing of memory. Fuster et al. (1985) demonstrated that a hypothermic PFC reduced performance of delayed memory, which supports our results. Furthermore, the PFC can be divided into the dorsolateral prefrontal cortex (DLPFC), at or near Brodmann’s areas (BA) 9/10 and 46, and the ventrolateral prefrontal cortex (VLPFC), at or near BA 6/8, 44, 45, and 47, according to their cognitive functions and anatomical structure (Ranganath, 2006). In our study, the pars opercularis and pars triangularis of the inferior frontal gyrus were a part of the VPFC and the frontal part of left middle frontal gyrus belonged to the DLPFC. The VLPFC is activated in tasks that require transient encoding (Paller and Wagner, 2002), organizing, reordering (Blumenfeld, 2006), and maintaining relevant items (Ranganath et al., 2004) and inhibiting distractions (Konishi et al., 1999). More specifically, the area near BA 6 and 8 is involved in encoding spatial items (Courtney et al., 1998), the area near left BA 45 and 47 is activated during classification and comparison of semantic items (Wagner et al., 2001b), the area near BA 44 and 47 is engaged during non-verbal memory (Braver et al., 2001), and that near BA 44 and 45 is involved in resisting distractions (Thompson-Schill and D’Esposito, 1997). Moreover, the DLPFC is activated in memory tasks that require reasoning and relating (Kroger et al., 2002; Blumenfeld, 2006; Murray and Ranganath, 2007), especially in tasks involving reasoning abstract relation (Kroger et al., 2002; Badre and Wagner, 2004), encoding relationships between items (Davachi and Goldman-Rakic, 2001), and reordering relevant items (Blumenfeld, 2006). Taken together, these findings explain why extensive atrophy of both the VLPFC and DLPFC due to CAS can lead to a decline in general memory functions. These findings also offer theoretical support for our findings.

#### Associative Memory

We found that GM volumes of the frontal part of the left middle frontal gyrus were positively correlated with associative memory scores, which indicated that atrophy of this region leads to associative memory decline. In previous studies, the DLPFC has been shown to be involved in memory tasks that require relating and reasoning (Kroger et al., 2002; Blumenfeld, 2006). Blumenfeld (2006) reported that the DLPFC was activated in tasks requiring reordering items, and Wagner et al. (2001a) found that the DLPFC was involved in encoding relationships among relevant items. These findings explained why atrophy of the left middle frontal gyrus results in associative memory impairment. Although some studies have found that the middle temporal gyrus and hippocampus are also involved in associative memory (Diana et al., 2007; Mayes et al., 2007), Becker et al. (2015) found that atrophy of these regions due to aging was not severe and Brehmer et al. (2020) demonstrated that atrophy of the frontal lobe can predict memory function more precisely in older adults. Similarly, atrophy of the middle temporal gyrus and hippocampus may not have been severe enough, or alternatively, they may not be closely correlated with associative memory in CAS patients.

#### Semantic Memory

The GM volumes of the left pars opercularis were positively correlated with semantic memory scores, which suggested that GM atrophy may lead to semantic memory decline. The left pars opercularis is located in the posterior VLPFC, which has been shown to be involved in semantic memory processes (Wagner et al., 2001b; Murray and Ranganath, 2007). Poldrack et al. (1999) reported that the VLPFC is specifically activated in memory tasks requiring semantic processing, and the posterior/dorsal region is active in both semantic and phonological processing. In a review of previous studies, they found that the posterior/dorsal VLPFC is also involved in semantic generation, semantic decision, lexical tasks, and viewing the world (Poldrack et al., 1999). Additionally, Stebbins et al. (2002) reported that semantic memory decline caused by aging was related to the reduced function of the DLPFC. These studies support our findings and suggest that atrophy of the VLPFC may be a leading risk factor of semantic memory impairment in patients with CAS.

### 7T-MRI

Benefiting from ultra-high resolution, 7T-MRI has become more widely used in clinical work and scientific research. Wei et al. (2018) demonstrated that 7T-MRI could visualize the superficial temporal artery as well as DSA. Furthermore, Matsushige et al. (2016a) succeeded in visualizing the triple-layered microstructure of the giant aneurysm wall using 7T-MRI. They also applied 7T TOF-MRI to detect submillimeter-range microaneurysms that are invisible under DSA and 3T-MRI (Matsushige et al., 2016b). Furthermore, Lüsebrink et al. (2013) further found that 7T-MRI T1 images are more precise than 3T-MRI for segmentation for structural analyses. However, there is still some debate regarding structural analyses of 7T-MRI images (Seiger et al., 2015; Chen et al., 2017) and further studies are needed.

### Limitation

We analyzed the 7T-MRI data of 24 patients with asymptomatic severe CAS and 10 healthy controls. Although our sample size is the largest among previous reports, the absolute number of patients is still small, which may result in false positives or negatives. In addition, we only analyzed memory functions and did not investigate other cognitive functions. Finally, structural analysis of 7T-MRI images remains controversial at this stage. Despite using the best available correction and processing procedures, insufficiencies are still likely and further studies are required.

## Conclusion

Patients with asymptomatic severe CAS show greater memory impairment compared with healthy controls. Patients with asymptomatic severe unilateral CAS showed extensive GM atrophy in the frontal, temporal, and parietal lobes and in small parts of the occipital lobe in both the stenosis-side and the contralateral hemispheres. The areas were consistent with the anterior circulation region. GM atrophy of the stenosis hemisphere was more severe than that of contralateral hemisphere. Memory impairment in patients with asymptomatic severe CAS may be related to GM atrophy of the left inferior frontal and middle frontal gyri. Moreover, the atrophy of the anterior medial frontal gyrus may be responsible for the decline of associative memory, whereas atrophy of the posterior inferior frontal gyrus may contribute to the decline of semantic memory.

## Data Availability Statement

The datasets presented in this article are not readily available in order to protect the subjects’ privacy. Requests to access the datasets should be directed to YZ, yanzhang135@163.com.

## Ethics Statement

The studies involving human participants were reviewed and approved by the Ethics Committee of Beijing Tiantan Hospital, Capital Medical University. The patients/participants provided their written informed consent to participate in this study.

## Author Contributions

PW, RL, and YZ: conception and design. PW and RL: acquisition of data. PW: analysis and interpretation of data and drafting the article. HC, RL, ZZ, DZ, and YZ: technical, administrative, and material support. YZ: approving the final version of the manuscript on behalf of all authors. YZ: study supervision. All authors critically revised the article and reviewed the submitted version of the manuscript.

## Conflict of Interest

The authors declare that the research was conducted in the absence of any commercial or financial relationships that could be construed as a potential conflict of interest.

## Abbreviations

ANOVA: analysis of variance
CAS: carotid artery stenosis
CBF: cerebral blood flow
CMS: clinical memory scale
CSF: cerebrospinal fluid
CTA: computed tomography angiography
DLPFC: dorsolateral prefrontal cortex
DSA: digital subtraction angiography
FDR: false discovery rate
GE: gradient echo
GM: gray matter
MMSE: mini-mental state examination
MQ: memory quotient
MRI: magnetic resonance imaging
PFC: prefrontal cortex
ROI: region of interest
TIA: transient ischemic attack
TIV: total intracranial volume
TPM: tissue probability map
VBM: voxel-based morphometry
VLPFC: ventrolateral prefrontal cortex
WM: white matter.
